# Silk fibroin-based biomaterials for cartilage/osteochondral repair

**DOI:** 10.7150/thno.74548

**Published:** 2022-07-04

**Authors:** Ziyang Zhou, Jin Cui, Shunli Wu, Zhen Geng, Jiacan Su

**Affiliations:** 1 Institute of Translational Medicine, Shanghai University, Shanghai, 200444, China; 2 Musculoskeletal Organoid Research Center, Shanghai University, Shanghai, 200444, China; 3 School of Medicine, Shanghai University, Shanghai 200444, China; 4 School of Life Sciences, Shanghai University, Shanghai 200444, China; 5 Department of Orthopedics Trauma, Changhai Hospital, Second Military Medical University, Shanghai, 200433, China; 6 School of Environmental and Chemical Engineering, Shanghai University, Shanghai 200444, China

**Keywords:** Silk fibroin, Tissue engineering, Biomaterials, Cartilage repair, Osteochondral repair.

## Abstract

Osteoarthritis (OA) is a common joint disease with a high disability rate. In addition, OA not only causes great physiological and psychological harm to patients, but also puts great pressure on the social healthcare system. Pathologically, the disintegration of cartilage and the lesions of subchondral bone are related to OA. Currently, tissue engineering, which is expected to overcome the defects of existing treatment methods, had a lot of research in the field of cartilage/osteochondral repair. Silk fibroin (SF), as a natural macromolecular material with good biocompatibility, unique mechanical properties, excellent processability and degradability, holds great potential in the field of tissue engineering. Nowadays, SF had been prepared into various materials to adapt to the demands of cartilage/osteochondral repair. SF-based biomaterials can also be functionally modified to enhance repair performance further. In this review, the preparation methods, types, structures, mechanical properties, and functional modifications of SF-based biomaterials used for cartilage/osteochondral repair are summarized and discussed. We hope that this review will provide a reference for the design and development of SF-based biomaterials in cartilage/osteochondral repair field.

## 1. Introduction

Osteoarthritis (OA) is a degenerative disease, which involves the whole joint (including articular cartilage, subchondral bone, ligaments, joint capsule, synovium, and muscles around the joint) 1. The pathogenesis of OA is complex and its prevalence is correlated with age, gender and obesity status 2, 3. Patients suffer from severe pain and even amputation caused by OA, resulting in a sharp decline in quality of life 4. At present, about 250 million people around the world are suffered from OA 5. With the aging of the global population, increasing obesity, and joint injuries, the number will increase by about 50% in the next decade 6. Statistically, OA costs about $303 billion a year in medical expenses and lost income, which is a massive burden on suffered individuals, health systems, and the wider socio-economy 7. Typically, OA will occur when the dynamic balance between cartilage destruction and repair is broken, due to excessive mechanical load and pathological factors 8-11. Furthermore, untimely repair of cartilage defects can lead to further deterioration of OA 12, 13. Conversely, under osteoarthritic conditions, the rising activity of multiple proteases gives rise to augmented cartilage destruction 14. In brief, the development of OA and cartilage destruction is a vicious cycle. Therefore, cartilage repair is the essential element in the treatment of OA 15, 16.

Currently, the treatment strategies of cartilage repair include microfracture (bone marrow stimulation), autologous chondrocyte transplantation, and allogeneic/autologous cartilage transplantation 17, 18. Although these treatment strategies are widely used in the clinic, they still have several shortcomings. For example, microfractures often cause the formation of fibrocartilage 19. In addition, the application of allogeneic/autologous cartilage transplantation is limited by donor shortage, poor integration, and surgical infection 20, 21. Recently, tissue engineering has become a promising strategy for repairing damaged tissue, such as skin, heart, and bone 22-24. In general, cartilage defects are caused by trauma, long-term wear, disease, and aging. The self-repairing ability of cartilage is limited due to the absence of blood vessels and the poor chondrocytes proliferation 25, 26. Therefore, introducing nutrients or stem cells from adjacent tissues is an effective strategy to accelerate cartilage repair 27. Subchondral bone is adjacent to and underneath the cartilage and is rich in vascular tissue and a variety of stem cells 28-30. Therefore, constructing osteochondral defects (so that nutrients and a variety of stem cells in the subchondral bone can arrive at the cartilage region) to achieve integrated osteochondral repair is expected to accelerate cartilage repair 31, 32.

**Figure 1:**
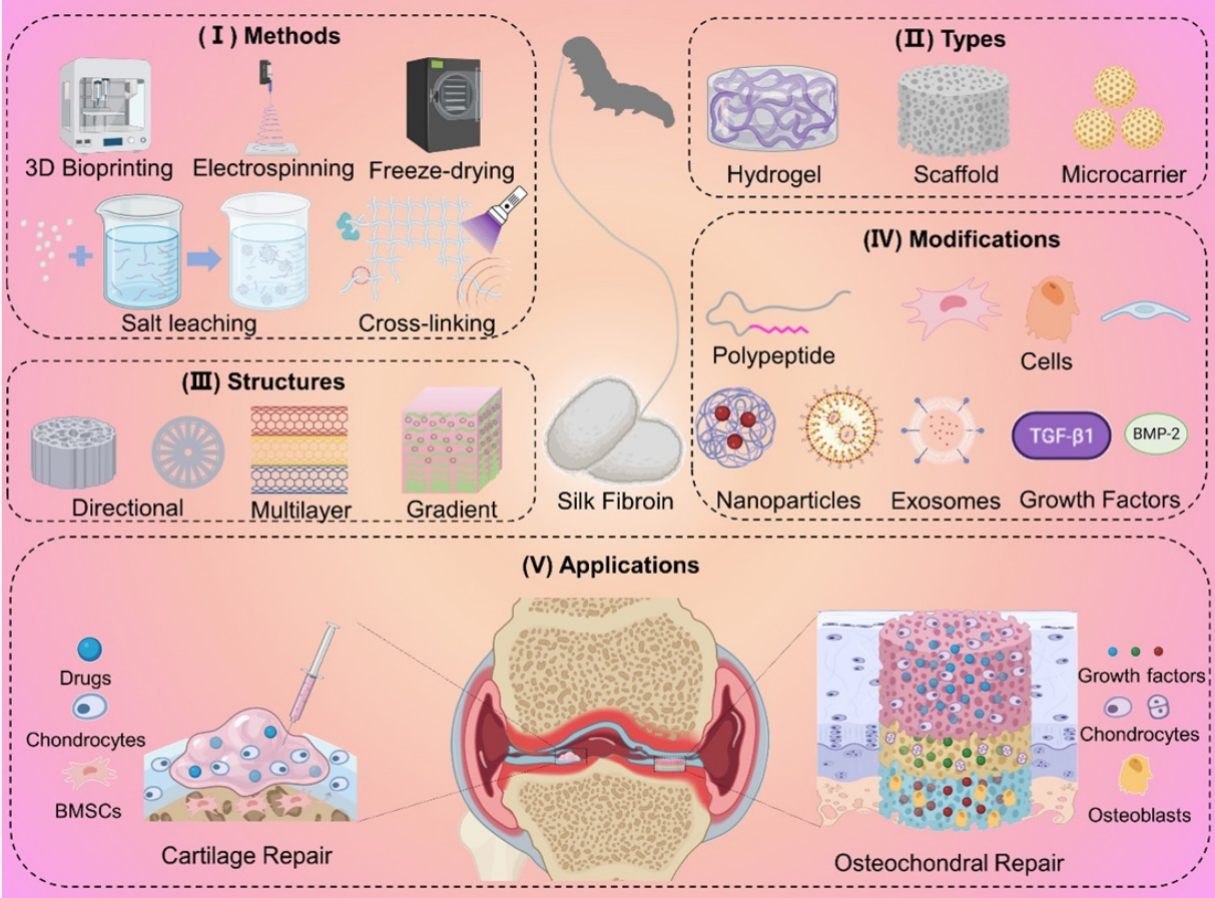
Preparation methods, materials types, structures, and modifications of SF-based biomaterials for cartilage/osteochondral repair. (I) Preparation methods including 3D bioprinting, electrospinning, and freeze-drying. (II) Representative types including hydrogel, scaffold, and microcarrier. (III) Directional structure, multilayer structure, and gradient structure mimic natural cartilage or subchondral bone tissue. (IV) Functional modification of SF-based biomaterials by nanoparticles, exosomes, cells, polypeptides, and growth factors. (V) The application of SF-based biomaterials for cartilage/osteochondral repair.

Silk fibroin (SF) is a kind of natural protein material that has been used in the clinic for decades. Compared with synthetic and natural materials, SF exhibits superior toughness, biocompatibility, biodegradability, and thermal stability. Additionally, SF can also significantly promote bone and cartilage regeneration 33, 34. Based on these, SF-based biomaterials have been widely studied and used in the biomedical field, especially in tissue engineering 35. Moreover, SF-based biomaterials have more incomparable advantages than other materials in the cartilage/osteochondral repair, including: (1) Excellent cytocompatibility (suitable growth environment could accelerate cell proliferation and differentiation); (2) unique mechanical property (cell adhesion and growth require adequate mechanical support); (3) non-toxic degradation products and controllable biodegradability (the space left by the degradation of biomaterials is essential for the growth of regenerative tissues); (4) desirable processability (SF-based biomaterials are satisfactory for a variety of fabricating methods and modifications) 36, 37. Therefore, SF-based biomaterials have great research value and application prospects in the field of cartilage/osteochondral repair (**Figure 1**).

Herein, we review the research of SF-based biomaterials in cartilage/osteochondral repair in the past decade. We hope this review can provide a theoretical basis for the design and preparation of SF-based biomaterials for cartilage/osteochondral repair.

## 2. Silk fibroin: source, composition, structure, and features

**Figure 2:**
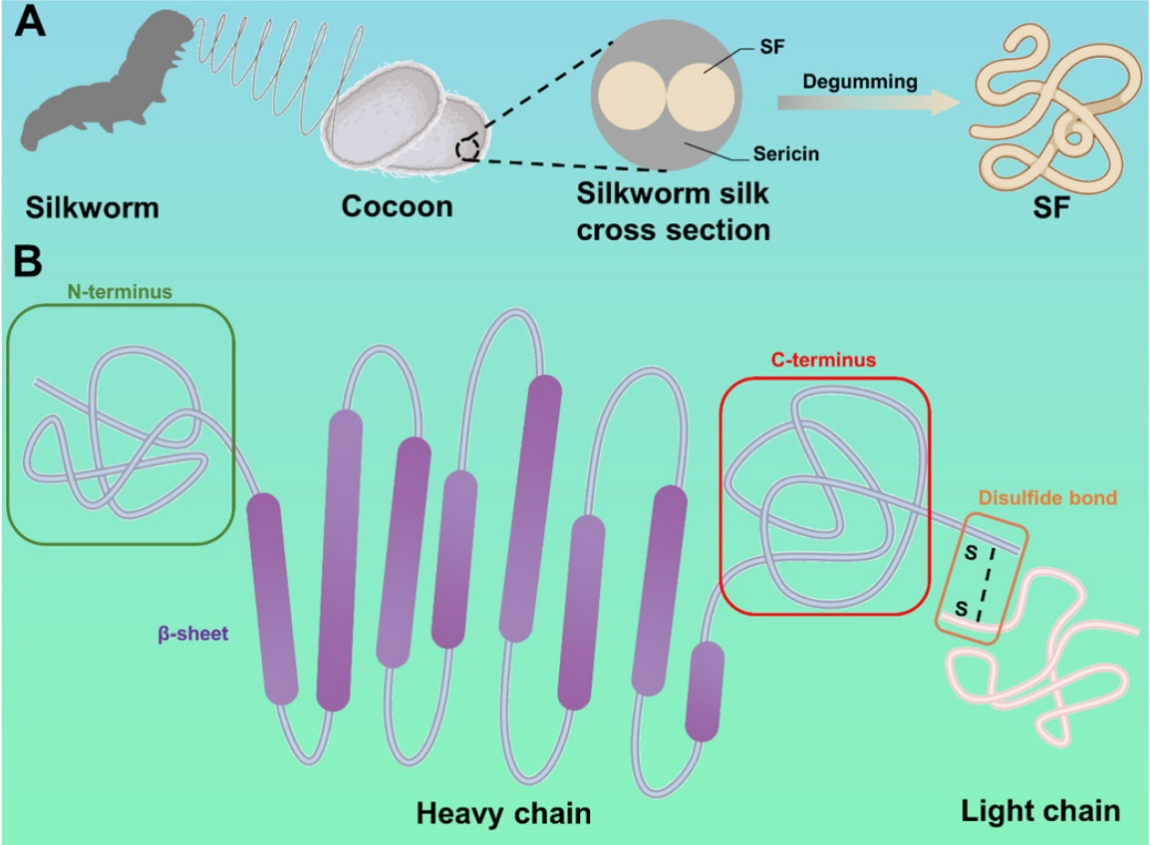
Schematic illustration of the SF: (A) The source of SF. (B) The composition and structure of SF. SF consists of heavy chain and light chains connected by disulfide bonds.

Generally, silk is secreted by various animals, such as silkworms, spiders, scorpions, mites, and bees. Compared with other silk, silkworm silk is the only silk that can be produced on a large scale and has been applied in the clinic. Hence, this review focuses on the description and discussion of silkworm silk. As shown in **Figure 2A**, a silkworm silk consists of two SF fibers coated with sericin. To obtain pure SF, silkworm silk needs to be degummed, rinsed, and dried 38.

SF has a complex molecular structure, which is composed of disulfide bond-linked light chain (molecular weight 26KDa) and heavy chain (molecular weight 390KDa), as shown in **Figure 2B** 39. The light chain, which has excellent elasticity, is formed of disordered amino acid sequences and does not participate in the formation of crystal structure. The heavy chain consists of several repetitive sequences, including Gly-Ala-Gly-Ala-Gly-Ser sequences, Gly-Ala-Gly-Ala-Gly-Tyr sequences, and Gly-Ala-Gly-Ala-Gly-Ser-Gly-Ala-Ala-Ser sequences. The heavy chain has remarkable tensile strength due to these sequences. Therefore, attributing to the heavy and light chains, SF has better mechanical properties than other natural and synthetic materials (maximum fracture elongation, ultimate Young's modulus, and toughness are 4%, 26%, 300-700MPa, and 70-78MJ/m3, respectively) 40. In addition, the mechanical properties of SF can be adjusted by changing the size, quantity, orientation, and arrangement of crystalline (silk Ⅱ) and amorphous structures.

The chemical composition and structure of SF endow it with excellent biocompatibility and biodegradability. The degradation products of SF (mainly amino acids and peptides) not only have no cytotoxicity, but also can provide nutrition for tissue regeneration. This makes SF have a good prospect of clinical application 40. Actually, surgical sutures and wound dressings prepared by SF have been widely used in biomedical fields. Currently, SF is attracting more and more attention in the field of cartilage/osteochondral repair 41.

## 3. Articular cartilage and subchondral bone

The articular cartilage is a multilayer tissue, including the hyaline cartilage layer and calcified cartilage layer. In addition, the subchondral bone is located directly below the articular cartilage (**Figure 2**). Hyaline cartilage, calcified cartilage, and subchondral bone form a massive complex with different components, structures, and properties 42.

Hyaline cartilage is a non-blood supply tissue composed mainly of water, type II collagen and aggregates 43. The core protein of aggrecan has covalent binding and powerfully negatively charged glycosaminoglycan side chain. It is non-covalently linked to hyaluronic acid through connexin to form a proteoglycan polymer 44. Highly cross-linked type II collagen fibers form an organized network that captures negatively charged proteoglycan aggregates and interacts with other collagen, small proteoglycan, and other cartilage-specific/non-specific matrix proteins 45. Specially, chondrocytes are the only cell type in cartilage, accounting for only 1-2% of the total volume of cartilage 46. However, chondrocytes maintain basic metabolism and proliferate slowly due to the insufficient mitotic activity of mature articular chondrocytes 47.

Calcified cartilage is a thin layer of tissue between hyaline cartilage and subchondral bone. It is produced by endochondral ossification and still exists after the growth plate is closed. Calcified cartilage is denser and more mineralized than the adjacent cartilage. Besides, there is a histologically defined tidal mark (tidemark) between calcified cartilage and articular cartilage 48. In addition, the biological composite formed by calcified cartilage and subchondral bone is especially suitable for transmitting and distributing mechanical force under physiological load. Compared with cartilage, most of the mechanical load is borne by the composite.

Subchondral bone is located directly below the calcified cartilage with unique structural, biological, and mechanical properties 49. It forms a plate-like structure similar to cortical bone in other skeletal parts. In comparison to cortical bone, subchondral bone has more porous and metabolic activity. In addition, the bones in the subchondral bone layer combine to form a trabecular network (also known as cancellous bone). The trabeculae in cancellous bone are oriented in different directions according to their location, and they provide a unique structural network to adapt to local mechanical effects. Moreover, the periosteum forming the edge of the joint is in direct contact with the joint capsule and synovium. Under the joint capsule and synovium, ligaments and tendons are inserted into the bone to form a unique structure at the end 14.

In brief, hyaline cartilage, calcified cartilage and subchondral bone have different characteristics. It is worth noting that the species of silkworm affects the structure of SF molecule, and then affects the biological properties of SF-based biomaterials 50. For example, Saha et al. compared the performance of mulberry SF and non-mulberry SF in the field of bone and cartilage repair 51. Under similar conditions, they found that mulberry SF was more osteoinductive, while non-mulberry SF was more chondroinductive. Meanwhile, Singh et al. compared cartilage repair properties of mulberry SF hydrogels and non-mulberry SF hydrogels 34. Compared with mulberry SF hydrogels, non-mulberry SF hydrogels showed better-promoting effects on sulfated glycosaminoglycans and collagen deposition. This proved that non-mulberry SF is more suitable for cartilage repair. Additionally, the characteristics of hyaline cartilage, calcified cartilage and subchondral bone should be taken into account in the design of SF-based biomaterials for cartilage/osteochondral repair. For the cartilage layer, there is only one kind of chondrocyte with a gradient distribution. On the one hand, the SF-based biomaterial should be modified to promote the proliferation and differentiation of chondrocytes. On the other hand, the SF-based biomaterial should be endowed with a gradient structure to ensure the gradient distribution of chondrocytes (similar to primary cartilage). For the calcified cartilage layer, SF-based biomaterials should be designed as dense structure. The dense structure could prevent the migration of angiogenic cells from the subchondral bone layer to the cartilage layer and avoid cartilage layer fibrosis. For the subchondral bone layer, the biomineralization and antibacterial ability of SF-based biomaterials could be enhanced by adding metal ions 52-55. It is worth mentioning the synergistic role of osteogenesis and angiogenesis in promoting subchondral bone repair 56. Therefore, SF-based materials could add angiogenic factors to accelerate the repair of subchondral bone.

## 4. The application of SF-based biomaterials for cartilage/osteochondral repair

### 4.1. Preparation methods of SF-based biomaterials

#### 4.1.1. 3D bioprinting

3D bioprinting is a controllable rapid prototyping and additive manufacturing technology 57, 58. It allows personalized adjustment of size parameters and layer-by-layer printing of biomaterials with complex structures via computer assistance 59. In addition, 3D printed biomaterials show great potential in meeting the mechanical, structural and biological requirements for cartilage/osteochondral repair 60-62. Generally, printing equipment (biological printer) and ink (biological ink) are the two main components of 3D bioprinting technology.

**Figure 4:**
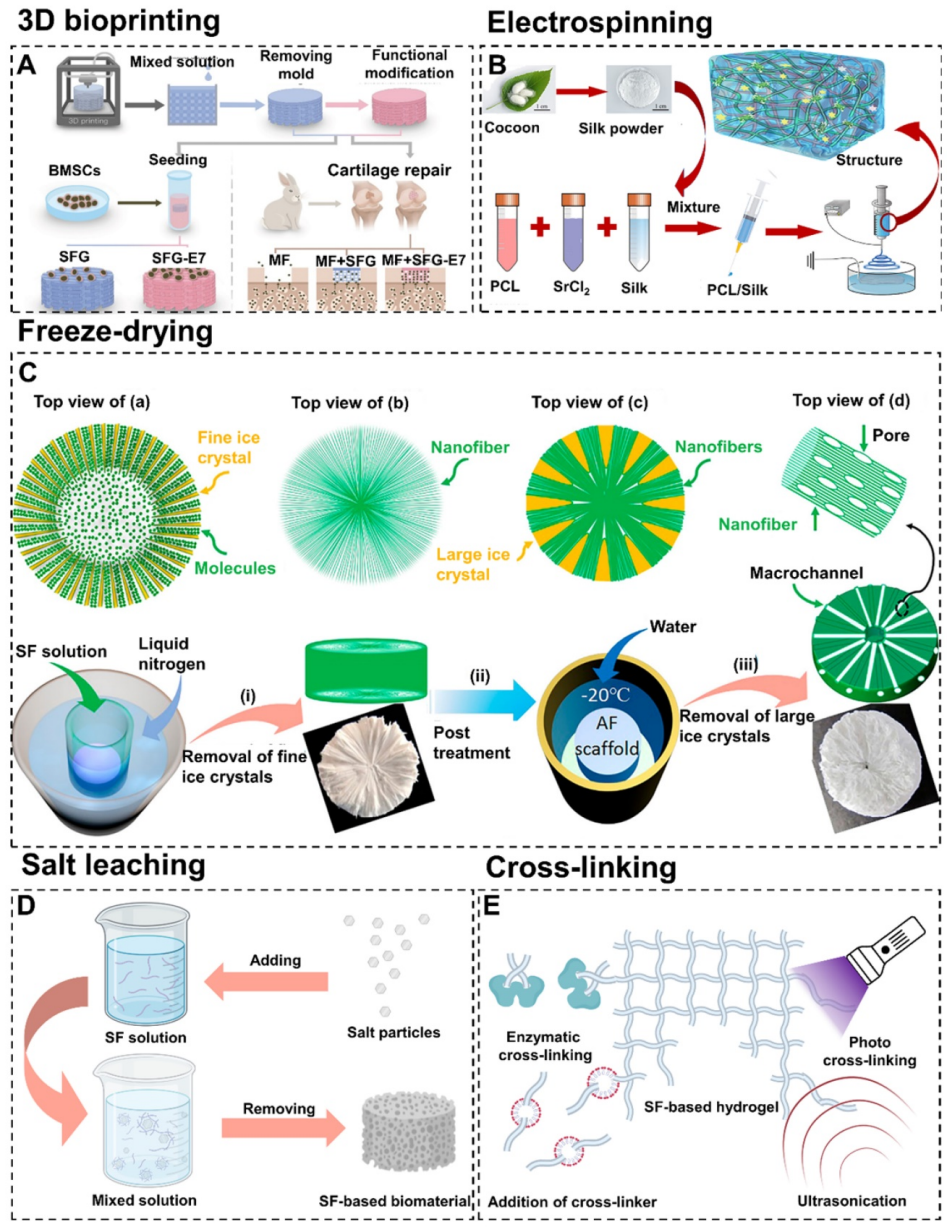
Preparation methods of SF-based biomaterials. (A) 3D bioprinting: schematic diagram of preparing SFG-E7 scaffolds by mixing SF and gelatin. Adapted with permission from 65, copyright 2017, WILEY-VCH Verlag GmbH & Co. KGaA, Weinheim. (B) Electrospinning: schematic diagram of preparing SP-Sr scaffolds by electrospinning PCL, SF, and strontium mixed solution. Adapted with permission from 66, copyright 2020, Elsevier Ltd. (C) Freeze-drying: fabricating 3D SF scaffolds with radially co-aligned nanofibers and interconnected macro-channels by a facilely guided ice-crystal growth and nanofiber assembly strategy. Adapted with permission from 67, copyright 2018, American Chemical Society. (D) Schematic diagram of fabricating SF-based biomaterials by salt leaching method. (E) Schematic diagram of preparing SF-based hydrogel prepared by various cross-linking methods.

Currently, the main types of 3D bioprinting equipment include inkjet printing, extrusion printing, and laser-assisted printing. Among them, the extrusion strategy is suitable for a wide range of materials and curing methods. Furthermore, compared with the inkjet printing method and laser-assisted method, the extrusion printing technology is able to process higher-resolution patterns and is more cost-effective 63. Additionally, extrusion 3D printing can effectively overcome the problem that SF-based biomaterials fail to be commercialized due to the insufficiency of standardization and industrialization in the preparation process. For instance, Trucco et al. constructed and verified the analytical model of a 3D extrusion bioprinting system based on extrusion to predict the width of deposited SF filaments 64. The model took into account the key printing process parameters (pressure and speed of biological ink) to predict the SF filament width from the various rheological properties of different hydrogels. This model helped researchers to define the most appropriate SF printing parameters to maximize the fidelity of the manufactured structure to the design parameters. In addition, in order to improve the affinity of SF-based biomaterials for chondrogenic cells, other natural macromolecular materials and stem cell-specific affinity peptides can be added when printing SF-based biomaterials. For example, Thunsiri et al. prepared bilayer bioactive biomaterial scaffolds with polylactic acid (PLA), polycaprolactone (PCL), hydroxyapatite (HA), chitosan (CS), and SF by 3D extrusion bioprinting 68. The experimental results showed that the scaffolds had the potential to treat full-thickness articular cartilage defects. Meanwhile, Shi et al. constructed SF-gelatin-E7 (SFG-E7) scaffolds by integrating SF with gelatin in combination with bone mesenchymal stem cell (BMSC)-specific-affinity peptide using 3D bioprinting technology (**Figure 4A**) 65. They found that E7 BMSCs specific affinity peptides significantly promoted the proliferation and differentiation of BMSC and the production of extracellular matrix (ECM). These works proved that extrusion 3D bioprinting had strong controllability and laid a foundation for industrialized and standardized production of SF-based biomaterials.

According to the reports, choosing the appropriate biological ink is the key to successful biological printing 69. Biological inks need to meet several basic standards, such as excellent bioactive, appropriate viscosity, suitable flexibility and great stability 70, 71. Generally, modification of SF biological ink is required to strengthen the bioactivity of SF when preparing SF-based biomaterials using 3D bioprinting. For example, Li et al. fabricated SF-based biological ink with platelet-rich plasma (PRP), and used 3D bioprinting technology to prepare SF-PRP scaffolds. The addition of PRP effectively enhanced the bioactivity of SF-based biological ink and accelerated cartilage repair 72. However, SF-based biomaterials still face the problem that the mechanical strength is inadequate to meet the needs of cartilage/osteochondral repair. In order to overcome this problem, SF biological ink can be modified with dual functions. For instance, Deng et al. designed SF-parathyroid hormone (SF-PTH) biological ink and mechanically modified SF-methacrylic anhydride (SF-MA) biological ink 73. Then, successfully constructed the integrated gelatin methacryloyl (GM) + SF-PTH/GM + SF-MA biphasic scaffold with excellent biological activity and a mechanical gradient via 3D bioprinting technology. In addition, *in vivo* experiments showed that the scaffold had desirable osteochondral repair ability.

#### 4.1.2. Electrospinning

Electrospinning is a highly controllable technology that allows fine-tuning of multiple parameters. Compared with other fiber spinning processes, electrospinning is able to produce long fibers with a smaller diameter and a higher ratio of surface area to volume. In addition, electrospun scaffolds prepared by assembling electrospun fibers have the advantages of low cost, simple process, and similar nanofibrous structure to ECM with interconnected pores compared with other tissue scaffolds 74. Moreover, due to the highly controllable of electrostatic spinning technology, it is available to prepare SF-based scaffolds with a wide range of pore sizes 75. For example, Huang et al. prepared SF scaffolds with gradient pore size under appropriate relative humidity by adjusting fiber diameter (concentration of electrospinning solution) and collector temperature 76. The experimental data showed that the large pore layer scaffolds with 37.2±12.9 μm pore size significantly promoted the migration of cells into the scaffold. The medium pore layer scaffold with 11.6±1.4 μm pore size was more beneficial to cell proliferation. Additionally, the small pore layer scaffold with a micropore size of 5.9±1.4 μm had higher mechanical properties.

In recent years, electrospinning has been widely used in the field of cartilage tissue engineering 77, 78. For instance, Li et al. constructed acellular three-dimensional meniscus scaffolds composed of PCL, SF, and strontium by wet electrospinning (**Figure 4B**) 66. The functional scaffolds had been successfully used for meniscus regeneration in the rabbit meniscectomy model. This study suggested that electrospinning could mix a variety of substrates and combine the characteristics of substrates to fit the needs of cartilage tissue engineering. Furthermore, SF-based scaffolds for full-thickness repair of hyaline cartilage, calcified cartilage and subchondral bone could also be prepared by electrospinning technique. For example, Liu et al. designed and synthesized CS/SF/hydroxyapatite three-layer scaffolds via electrospinning 79. Results confirmed that this CS/SF/hydroxyapatite three-layer scaffolds could mimic the chondral layer, calcified layer, and subchondral bone layer respectively to promote osteochondral repair. Generally, ordinary electrospinning usually produces two-dimensional films with tightly packed layers which hinder osteoid cells migration. To break through this limitation, electrospinning could be combined with other preparation methods to improve the cartilage/osteochondral repair effect of SF-based biomaterials. For instance, Chen et al. prepared three-dimensional/hyaluronic acid/SF scaffold (3DHAS) in a stepwise design by combining electrospinning, gas foaming and freeze-drying techniques 80. They found that 3DHAS had the characteristics of low density, large pore size, high porosity, strong water absorption capacity, and good mechanical stability. Importantly, there was excellent cartilage regeneration after subcutaneous transplantation of 3DHAS. Moreover, electrospinning could maintain the elasticity of SF (which is essential for cartilage/osteochondral tissue engineering) 81. For instance, Christakiran et al. reported a simple, scalable, and repeatable strategy to develop electrospun double-layer bioglass/SF composites for the repair of osteochondral defects 82. The biphasic composites exhibited both an elastic region pertinent for cartilage tissue and a stiff compression resistant region simulating the bone phase.

#### 4.1.3. Freeze-drying

Freeze-drying is a flexible and controllable technology for the preparation of biomaterials. Freeze-drying has attracted wide attention because it could control the growth of ice nuclei to adjust the pore size of biomaterials 83, 84. This method has shown certain advantages, such as process adaptability, excellent pore-making capacity, and environmental friendliness 85.

It is reported that the pore shape and orientation of biomaterials can be controlled by adjusting the freezing direction 86, 87. For example, Fan et al. prepared biomimetic anisotropic 3D SF scaffolds with the radial channel via directional freeze-drying technology (**Figure 4C**) 67. During the preparation process, heat is transmitted from the center to all around. Therefore, the growth direction of ice crystals is from the periphery to the center. Compared with other SF scaffolds, the freeze-drying scaffolds had a higher cell capture and growth promotion ability. More importantly, the scaffolds also regulated cell migration, arrangement, elongation, and interaction. In addition, the unidirectional pore could be prepared by changing the heat transfer direction from radial direction to the single direction via freeze-drying technology. For instance, Zhang et al. fabricated biomimetic SF-based scaffolds with cartilage ECM-like configurations (horizontally aligned structure of the surface layer, vertically aligned structure of the bottom layer and random pore structure of the middle layer) by directional freezing 88. The above two examples showed that freeze-drying technology can control the morphology of SF-based biomaterials. The radial pore structure and directional pore structure will be discussed in detail in* 4.3.1. Directional structure*.

Notably, the process of preparing biomaterials by freeze-drying technique causes the solvent to freeze and then sublimate, while having little effect on the solute. Therefore, freeze-drying is also convenient for SF-based biomaterials to carry drugs and growth factors 89. For instance, Sun et al. successfully prepared oriented microtubule collagen/CS/SF-transforming growth factor-β1 (TGF-β1) scaffolds with good hydrophilicity by freeze-drying technique 90. The scaffolds had good physical and chemical properties, mechanical properties, and biocompatibility. Furthermore, the scaffolds induced cartilage and subchondral bone regeneration by releasing TGF-β1.

#### 4.1.4. Salt leaching

Salt leaching is widely used to prepare SF-based biomaterials due to its high effectiveness and low cost. This method induces SF aggregation by adding salt particles to the SF solution. Porous SF biomaterials can be obtained by removing the undissolved salt particles (**Figure 4D**) 91. The properties of SF-based materials fabricated by salt-leaching are influenced by the size of salt particles and the concentration of SF solution 92. In general, salt-leaching is combined with other methods to fabricate SF-based biomaterials. For example, Ribeiro et al. prepared a novel SF-based material by combining salt-leaching and freeze-drying methods 93. This material had suitable porosity and mechanical properties for cartilage repair. In addition, Zhou et al. prepared a SF-CS material by combining salt-leaching and cross-linking methods 94. This material could maintain the chondrocyte phenotype, attenuate the interleukin (IL)-1β-induced inflammatory response of chondrocytes *in vitro*, and accelerate osteochondral repair *in vivo*.

#### 4.1.5. Cross-linking

As mentioned in section 2, SF contains hydrophobic residue repetitive sequences and a large number of polar groups 95. Therefore, SF has excellent cross-linking properties. The commonly used cross-linking methods for preparing SF-based hydrogels include enzymatic cross-linking, addition of cross-linker, photo cross-linking and ultrasonication (**Figure 4E**). Enzymatic cross-linking is a chemical cross-linking method which induce cross-linking reaction through activating certain groups by utilizing the catalysis of enzymes. This method can maintain the mechanical properties and biocompatibility of SF due to its mild reaction condition. For example, Zhang et al. constructed a novel enzymatically cross-linked SF-laponite (LAP) nanocomposite hydrogel 96. This hydrogel had good biocompatibility and excellent mechanical properties. Both the *in vitro* and *in vivo* experiments showed that this hydrogel could stimulate osteogenesis and chondrogenic differentiation of BMSCs, and accelerate the regeneration of osteochondral defects. Meanwhile, Li et al. fabricated a SF-tyramine-substituted gelatin (GT) hydrogel by combining enzymatic cross-linking method and 3D bioprinting 97. This hydrogel had good biocompatibility and could be used as a cell delivery vehicle to accelerate cartilage repair. However, the high price of enzyme limits the development of enzymatic cross-linking. Cross-linkers are a series of substances that can bond multiple linear molecules into a network structure. In addition, cross-linkers are cheaper than enzymes and can enhance the mechanical properties of SF-based hydrogels by forming stable covalent bonds. For example, Wang et al. constructed a SF/diglycidyl ether (BDDE) hydrogel with high elasticity and mechanical stability by using BDDE as cross-linker 98. The hydrogel had remarkable biocompatibility and the ability of long-term retention in the articular cavity. Photo cross-linking refers to control the cross-linking process through using ultraviolet, visible light or gamma rays by adding photo-initiators. Compared with other methods, the photo cross-linking has the fastest cross-linking rate. For instance, Piluso et al. prepared a cell-laden SF hydrogel in a few minutes by using riboflavin as photo-initiators 99. This hydrogel showed excellent cytocompatibility with cartilage derived progenitor cells, mesenchymal stem cells, and dental pulp derived stem cells and was expected to be a promising tissue engineering scaffold material for cartilage repair. However, the residues of photo-initiators and cross-linkers may lead to the cytotoxicity of SF-based biomaterials 100. Ultrasonication is a physical cross-linking method. This method accelerates cross-linking by increasing local temperature and shear-force, which can avoid the cytotoxicity problems caused by photo-initiators and cross-linkers. For instance, Yuan et al. prepared an ultrasonication-induced SF hydrogel 101. This hydrogel showed satisfactory physicochemical and biomechanical properties and could promote cartilage regeneration.

### 4.2. Types of SF-based biomaterials

#### 4.2.1. Hydrogels

Hydrogel is a hydrophilic three-dimensional network polymer material formed by physical or chemical cross-linking 102-104. The physical properties of the hydrogels are similar to biological tissues, such as high water content, rubber texture, and low interfacial tension. Therefore, hydrogels have excellent biocompatibility with surrounding tissues due to their physical properties are similar to natural tissues. Additionally, hydrogels are ideal for imitating the complex and organized network of natural ECM 105, 106. Besides, the natural EMC-like microenvironments of hydrogels are suitable for loading cells to promote cartilage/osteochondral repair 107. For example, Cui et al. presented a rapid and cyto-compatible photo cross-linking process to produce cell-encapsulated SF hydrogels cross-linked Through the formation of di-tyrosine bonds 108. The high density of chondrocytes in this hydrogel effectively accelerated cartilage repair.

It was reported that the mechanical properties, shape, and swelling properties of hydrogels could change with the alternation of external conditions, such as temperature, pH value, and ion concentration 109. The environment-responsive hydrogel designed based on this characteristic can realize the intelligent release of chondrogenic and osteochondrogenic drugs 110, 111. For example, Xu et al. prepared a series of stimulus-responsive composite hydrogels using SF and CS as raw materials and 1-(3-Dimethylaminopropyl)-3-ethylcarbodiimide/N-Hydroxysuccinimide as cross-linking agent 112. The swelling properties of hydrogels showed stimulus-response under different pH and ion concentrations. This work indicated that hydrogels have strong plasticity.

**Figure 5:**
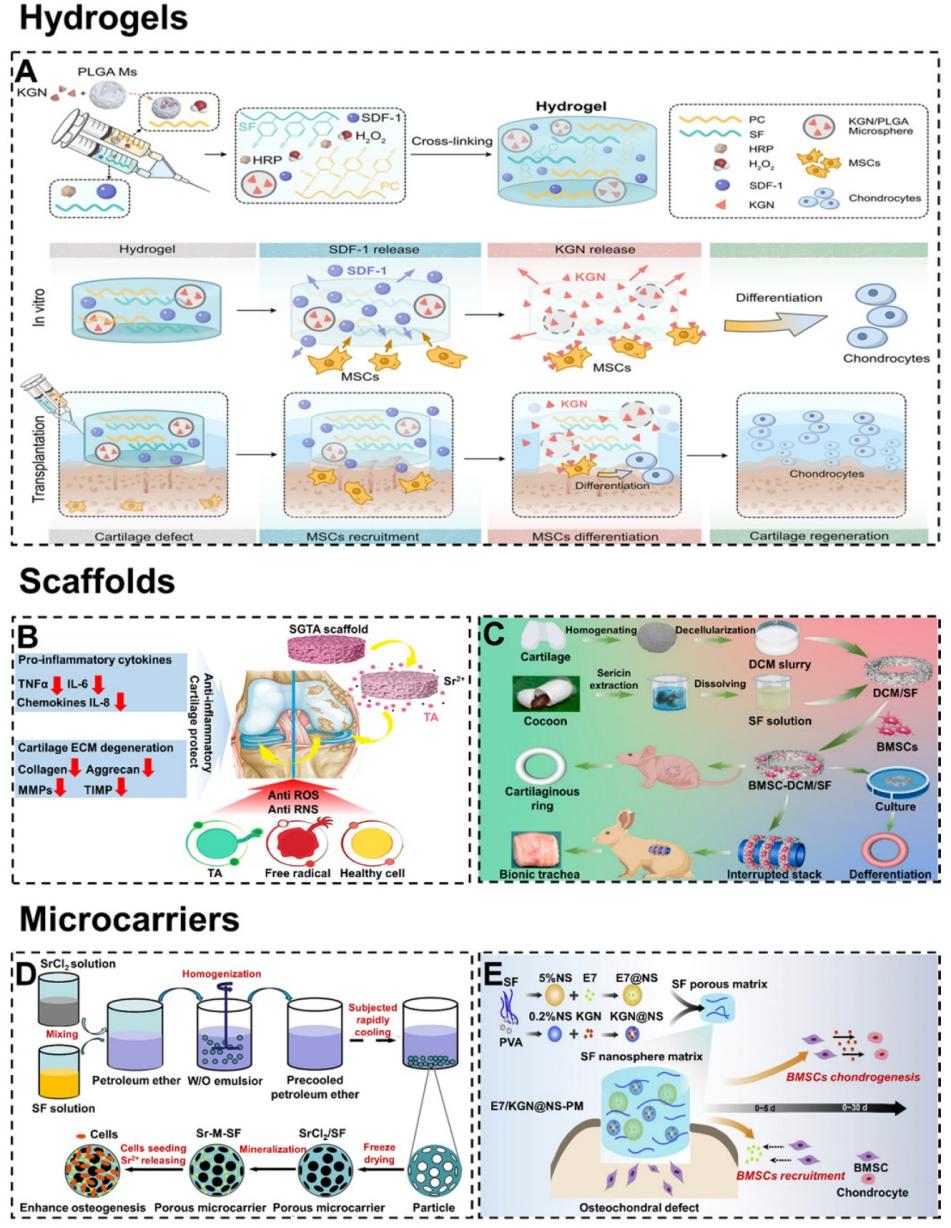
Representative types of SF-based biomaterials. Hydrogel: (A) Injectable PC-SF hydrogels loading with SDF-1, PLGA, and KGN promote recruitment and differentiation of stem cells for cartilage regeneration. Adapted with permission from 115, copyright 2021, Springer Nature. Scaffolds: (B) Mechanism of the SF/GO scaffolds delay OA and protect cartilage. Adapted with permission from 116, copyright 2021, Elsevier Ltd. (C) Fabrication of DCM/SF scaffold for repairing cartilaginous tissue. Adapted with permission from 117, copyright 2021, Elsevier Ltd. Microcarriers: (D) Schematic diagram of synthesizing Sr-M-SF porous microcarriers for enhancing osteogenesis. Adapted with permission from 118, copyright 2021, Elsevier Ltd. (E) Schematic diagram of producing SF nanospheres microcarriers for osteochondral repair. Adapted with permission from 119, copyright 2020, Elsevier Ltd

Importantly, the injectability exhibited by hydrogels is very practical in tissue engineering. This feature allows the hydrogel to fit tightly at the interface of cartilage defects to elevate the integration effect. Besides, injectable hydrogels as drug carriers adapt easily to defects of any size or shape and allow drugs to be evenly distributed 113, 114. For example, Dong et al. designed an injectable p-hydroxybenzene propanoic acid-modified chitosan/SF hydrogel with loading kartogenin (KGN)/poly(lactic-co-glycolic acid) microsphere (**Figure 5A**) 115. The hydrogel was injected *in situ* to recruit endogenous stem cells and induce them to differentiate into chondrocytes. In addition, after injection of the hydrogel into the cartilage defect site, there was a transition from solution to gel state. Unfortunately, the complicated procedures and toxicity of physicochemical transition methods still hinder further applications of injectable hydrogels. To overcome this problem, Yuan et al. developed an injectable SF hydrogel in a novel one-step ultrasonication cross-linking method 101. In this way, the toxic side effects caused by the gel transformation of injectable hydrogels in cartilage/osteochondral defects could be avoided.

#### 4.2.2. Scaffolds

Scaffolds are 3D porous matrices that promote cartilage/osteochondral repair and regeneration by providing a specific microenvironment. Ideally, scaffolds should be able to: (1) Promote transport of nutrients, and cell survival, proliferation, and differentiation; (2) provide suitable structural mechanical support; (3) degrade at a controllable rate; (4) present minimal inflammation and toxicity in vivo 120-123.

Nowadays, SF scaffolds have been widely used in cartilage/osteochondral engineering 124, 125. Compared with hydrogels, the scaffolds have fixed shapes and higher mechanical strength. In addition, the stable surface of the scaffolds could be modified by functional coating to improve cartilage/osteochondral repair performance 126, 127. For example, Li et al. prepared SF/graphene oxide (GO) meniscus scaffolds with tannic acid/Sr^2+^ functional coating (**Figure 5B**) 116. According to the results of tissue staining and the Osteoarthritis Research Society International scoring system, the expression of inflammatory cytokines (such as IL-6, IL-8, and MMPs) in the rat knee joint tissue was significantly down-regulated. Meanwhile, cartilage degeneration and OA injury were also inhibited. In addition, scaffolds could be designed into different 3D shapes to fit the notch of cartilage/osteochondral. For instance, Gao et al. have successfully fabricated porous decellularized cartilaginous matrix/silk fibroin (DCM/SF) scaffolds (**Figure 5C**) 117. It had been confirmed that DCM/SF scaffolds supported cartilage ring regeneration of BMSCs both in vivo and in vitro. Compared with DCM scaffolds and SF scaffolds, DCM/SF scaffolds significantly promoted the chondrogenesis of implanted BMSCs. In a rabbit model, the scaffold accomplished the restoration of the cricoid tracheal cartilage.

Scaffolds could serve as an adjunct to cell therapy to promote cell attachment, growth and differentiation. For example, Yang et al. synthesized CS/SF porous scaffolds loaded with C-type natriuretic peptide gene-modified BMSCs to accelerate the repair of articular cartilage defects 128. Generally, it is difficult to preserve scaffold-cell composites for a long time, which limits the applications of SF-based scaffolds. To address this difficulty, ran et al. developed a SF scaffold loaded with cartilage stem/progenitor cells 129. The SF scaffold-cell composites could be preserved in liquid nitrogen, and their activity, migration, and chondrogenic capacity were unaffected for up to 3 months.

#### 4.2.3. Microcarriers

Microcarriers usually refer to small spherical scaffolds suitable for cell culture, growth, and transport 130, 131. High specific surface area (promote cell growth and maintain cell differentiation phenotype) and injectability (realize tissue regeneration by directly injecting into the target site) enable microcarriers to accelerate cartilage/osteochondral repair 132, 133. In particular, porous microcarriers could provide a protected environment for seed cells attachment, proliferation, migration, nutrient exchange, and metabolic waste excretion 134. For example, Galuzzi et al. compared the activity of chondrocytes under the three models (pellet, alginate beads, and SF/alginate microcarriers) 135. The experiments proved that chondrocytes in the SF/alginate microcarriers were the most active and secreted the most ECM. Meanwhile, Fang et al. prepared strontium-containing SF porous microcarriers with the dual functions of bioactive factor and cell delivery (**Figure 5D**) 118. Cytobiological analysis showed that the porous microcarriers allowed attachment and proliferation of seeded cells. Besides, the released Sr^2+^ could stimulate the osteogenic differentiation of BMSCs.

To circumvent the problem of scarce cell sources, microcarriers could be loaded with growth factors to promote homing, adhesion, and growth of chondrogenic/osteogenesis cells 136. For instance, He et al. synthesized the SF/CS microcarriers by loading TGF-β1 to accelerated chondrogenesis 137. In addition, microcarriers could be loaded with active polypeptides and drugs to stimulate cartilage/osteochondral repair. For example, Zhang et al. prepared functional SF nanospheres microcarriers with peptides E7 (7-amino acid (EPLQLKM) small peptide) and KGN (**Figure 5E**) 119. The microcarriers rapidly release peptides E7 in the early stage to recruit BMSCs and provide relatively slow and sustained KGN release to induce cartilage formation of BMSCs, thus promoting osteochondral regeneration.

### 4.3. Structures of SF-based biomaterials

At present, different kinds of SF-based biomaterials for cartilage repair have their characteristics and advantages. It is worth mentioning that the role of the internal structure of SF-based biomaterials in cartilage/osteochondral repair cannot be ignored. Furthermore, SF-based biomaterials were designed in different structures, including directional structure, layered structure, and gradient structure. For cartilage/osteochondral tissue engineering, these elaborate structures were excellent in terms of improving the repair effects.

#### 4.3.1. Directional structure

**Figure 6:**
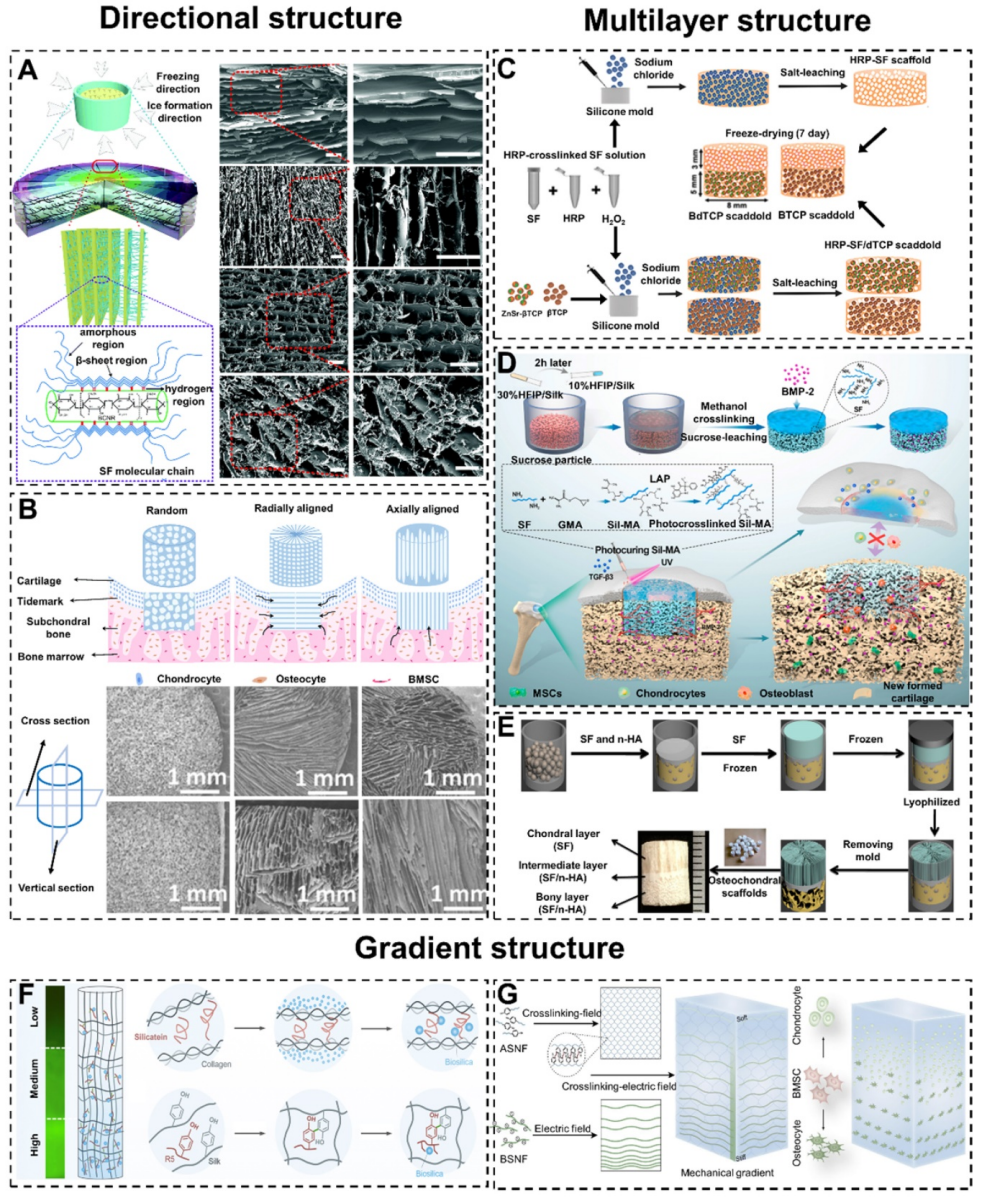
Mimic Cartilage/Osteochondral structures of SF-based biomaterials. Directional structure: (A) Formation mechanism of radial lamellae and intercalation structure, and the cross-section SEM images. Adapted with permission from 141, copyright 2017, The Royal Society of Chemistry. (B) Osteochondral repair of SF scaffolds with random structure, radially aligned structure, and axially aligned structure, and the cross section and vertical section SEM images. Adapted with permission from 142, copyright 2020, The Royal Society of Chemistry. Multilayer structure: (C) Schematic illustration of preparing BdTCP scaffold and BTCP scaffold in osteochondral regeneration. Adapted with permission from 143, copyright 2019, American Chemical Society. (D) Preparation and mechanism of the integral bilayer SF scaffold combined with Sil-MA hydrogel in osteochondral repair. Adapted with permission from 144, copyright 2021, Elsevier Ltd. (E) Preparation process of integrated three-layer osteochondral scaffolds. Adapted with permission from 145, copyright 2014, American Chemical Society. Gradient structure: (F) Synthesis procession of biomimetic GSSR5 composites: biosilica particles and R5 peptide self-assembly on SF to form gradient structure. Adapted with permission from 146, copyright 2017, Elsevier Ltd. (G) Schematic illustration of preparing SF hydrogels with gradient structure and the control of cell differentiation. Adapted with permission from 147, copyright 2020, Springer Nature.

In recent years, bioactive SF-based scaffolds with radial or axial pore structures have been developed to enhance cell migration and infiltration *in vivo* and *in vitro*, and further accelerate cartilage/osteochondral regeneration 138, 139. For example, Yang et al. combined SF and decellularized cartilage ECM by temperature gradient-guided thermal induced phase separation to produce composite scaffolds (axial pore structures scaffolds) 140. The results showed that the axial pore structures endowed the scaffolds with remarkable mechanical properties and were suitable for adipose stem cell attachment and proliferation. Meanwhile, Chen et al. prepared three-dimensional SF/bacterial cellulose nanoribbon (SF/BCNR) composite scaffolds with a radial layered structure by directional freezing technology (**Figure 6A**) 141. The radial interconnected pores promoted the flow of nutrients and waste to maintain cells vitality and provided robust mechanical performance. Therefore, the radial pore structures SF/BCNR scaffolds had high application potential in the field of cartilage/osteochondral regeneration.

Although there have been numerous studies demonstrating that the directional structure of scaffolds could accelerate cartilage/osteochondral regeneration. However, the underlying mechanism of directional structure promoting cartilage/osteochondral repair is still unclear. At present, only comparative studies between different structures are available. For instance, Feng et al. constructed three kinds of biomimetic SF/collagen composite scaffolds with different pore structures (random pore, radial pore, and axially arranged pore), and evaluated their ability to regenerate osteochondral defects in the rabbit model (**Figure 6B**) 142. The experimental data showed that the radial and axially arranged scaffolds had structural and mechanical anisotropy. Due to their high porosity, appropriate elastic modulus, good biocompatibility, and favorable three-dimensional microenvironment, these scaffolds enhanced cell activity in vitro and in vivo. Compared with random scaffolds, these properties contributed to a faster regeneration rate and better regeneration effect of osteochondral defects. Furthermore, in the comparison between radial pore structures and axial structures, the radial pore structures had a more significant effect on accelerating osteochondral repair.

#### 4.3.2. Multilayer structure

For cartilage/osteochondral tissue engineering, the key to the preparation of suitable scaffolds lies in the design of the structure 148. Meanwhile, scaffolds should closely simulate the physiological structure and environment of natural osteochondral tissue. In addition, on the basis of the physiological structure of natural subchondral bone and cartilage, many researchers have designed double-layer scaffolds with a cartilage layer and a subchondral bone layer to regenerate cartilage and subchondral bone at the same time 149-152. For example, Ribeiro et al. constructed a new type of double-layer composite scaffold with multiple functional modifications for osteochondral regeneration (**Figure 6C**) 143. The double-layer scaffolds included horseradish peroxidase-cross-linked SF (HRP-SF) cartilage layer, HRP-SF/ZnSr-doped β-tricalcium phosphate (β-TCP) subchondral bone-like layer (HRP-SF/dTCP layer).

They found that different layers in the bilayer scaffolds stimulated the expression of different genes in cells to promote the repair of osteochondral. In order to overcome the poor integration of regenerated tissue and primary tissue, Wu et al. constructed a kind of double-layer SF composite scaffolds with a peripheral package (**Figure 6D**) 144. The scaffold consisted of a dense and smooth biomimetic cartilage layer, a porous layer loaded with bone morphogenetic protein-2 (BMP-2) and a hydrogel coating loaded with transforming growth factor-β3 (TGF-β3). The experimental results showed that the double-layer SF scaffolds accelerated the regeneration of osteochondral, and the encapsulation hydrogel strengthened the integration of tissues. This special multi-layer design pointed out that researchers could achieve better cartilage/osteochondral repair by combining different types of SF-based biomaterials.

With the deepening of the study of double-layer scaffolds, the inherent defects in osteochondral repair become more apparent. The poor integration of the repaired cartilage layer and subchondral bone layer leads to a clear boundary between them 153, 154. As we mentioned earlier in *3. Articular cartilage and subchondral bone*, the calcified cartilage layer is an important physical barrier between cartilage and bone in different oxygen and nutrient environments. Without the calcified cartilage layer, the formation of regenerated cartilage and osteochondral tissue will be damaged 155. In order to closely simulate the natural osteochondral structure and regeneration environment, three-layer scaffolds have become available 156, 157. For instance, Ding et al. have successfully prepared biomimetic three-layer osteochondral scaffolds composed of SF/HA (**Figure 6E**) 145. Significantly, the intermediate layer could play a role in preventing the cells within the chondral and the bony layers from mixing with each other. Meanwhile, the intermediate layer enhanced the integration of articular cartilage and subchondral bone.

#### 4.3.3. Gradient structure

Many natural tissues, including bones, have multiple gradient structures to regulate cell behavior and guide tissue function 158-161. Generally, if biomaterials misfit with the structure of cartilage/osteochondral, the long-term repair effect will be unsatisfactory 162, 163. To mimic the progressive osteochondral structure, researchers could design SF-based scaffolds with gradient pore sizes 164, 165. For example, Xiao et al. designed and prepared SF/CS/nano-hydroxyapatite (NHA) osteochondral scaffolds with gradient pore sizes 166. The gradient pore size structure achieved better proliferation and differentiation of the stem cells to complete the repair of osteochondral defects.

The gradient modification of SF-based biomaterials could promote the gradient differentiation and distribution of cells to accelerate the repair of osteochondral 167. For example, Guo et al. prepared gradient silicified SF R5 peptide (GSSR5) composite biomaterials (**Figure 6F**) 146. The GSSR5 composite biomaterials regulated the osteogenic differentiation of hBMSCs in the bone induction environment. The material facilitated the differentiation of cells in a manner consistent with the R5 gradient distribution. This cellular differentiation and distribution consistent with native tissue facilitated osteochondral repair.

Generally, introducing gradient cues when preparing biomaterials requires special instruments and methods. Moreover, these instruments and methods are suitable for special materials only. To break through this limitation, Xu et al. prepared multifunctional building block hydrogels with tunable gradients using a simple low voltage electric field (**Figure 6G**) 147. This novel approach enables this hydrogel to exhibit multiple gradients. These gradient structures drove cells to differentiate in different directions and accelerated osteochondral repair.

### 4.4. Physical and chemical properties of SF-based biomaterials

#### 4.4.1. Mechanical properties of SF-based biomaterials

Articular cartilage is a special load-bearing tissue that provides friction-free movement in synovial joints such as the knee joint, hip joint, or shoulder. The elastic modulus of articular cartilage is between 1-10kPa, which requires the biomaterials to repair cartilage need to match its mechanical properties 168. Therefore, when preparing biomaterials for osteochondral repair, researchers should regulate the mechanical properties of SF 169. For example, Chameettachal et al. studied the effect of adding micronized CS into freeze-dried porous SF scaffolds 170. According to the experimental data, micronized CS significantly enhanced the mechanical properties of SF scaffolds when the ratio of SF to CS microparticles was 2: 1 (the compressive strength of the non-reinforced SF scaffolds and reinforced scaffolds for SF:CS=1:2, 1:1 and 2:1 were 14.40 ± 3.2, 25.40 ± 5.4, 28.10 ± 6.3 and 30.10 ± 7.5 MPa, respectively). Meanwhile, Mirmusavi et al. added multi-walled carbon nanotubes (MWNTs) based on CS/SF scaffolds 171. Characterization found that CS and MWNTs improved the tensile strength compared with pure SF scaffolds (the ultimate tensile strength of pure SF scaffolds, unsaturated CS/SF scaffolds, saturated CS/SF scaffolds and saturated CS-MWNTs/SF scaffolds were 28.32 ± 3.27, 39.21 ± 2.06, 41.18 ± 2.75 and 46.68 ± 2.20 MPa, respectively).

Normally, the articular cartilage is in a constant cycle of compression-rebound. In addition, during the process of cartilage/osteochondral repair, chondrocytes are more active in the dynamic environment. To prevent SF-based biomaterials premature damage after implantation in a dynamic environment of cartilage/osteochondral defect sites, SF-based biomaterials should exhibit excellent anti-fatigue ability 172. For instance, Huang et al. modified the SF with cholesterol or β-cyclodextrin, and prepared a kind of SF hydrogel with high mechanical strength (the compressive stress of SF hydrogels and modified-SF hydrogels were 0.1 and 3.16 MPa, respectively), high toughness and remarkable fatigue resistance (the modified-SF hydrogels remained in the original state without any deformation or strength degradation after 10 loading-unloading cycles at 60% strain) 173. Interestingly, the hydrogel could achieve self-repair after stress damage. This indicated that the self-repair hydrogels had great potential in cartilage/osteochondral repair.

#### 4.4.2. Degradation properties of SF-based biomaterials

The degradation properties of biomaterials directly affect the speed and quality of cartilage/osteochondral repair. Specifically, in the early stage of implantation, biomaterials should be stable to provide mechanical support for cells and tissues. After that, biomaterials need to be gradually degraded to match the new tissue growth 41.

As a kind of protein material, the degradation rate of SF-based biomaterials is mainly affected by proteases. Most proteolytic enzymes tend to degrade non-crystalline SF. This suggests that SF-based biomaterials with controlled degradability can be prepared by changing the content of crystalline structures 101. For instance, Jin et al. constructed water-stable SF films through increasing the content of crystalline structures by methanol treatment 174. Additionally, the degradation rate of SF-based materials can also be regulated by the introduction of proteinase inhibitors or protease binding sequence SF. For example, Pritchard et al fabricated a slowly degraded SF-based biomaterial by incorporating protein inhibitors 175. This material can realize the controlled release of drugs. On the contrary, Huang et al. prepared a novel kind of transgenic SF by introducing matrix metalloproteinase-2-sensitive sequences into SF 176. The transgenic SF had a quicker degradation rate and was non-toxic to BMSCs.

### 4.5. Functional modification of SF-based biomaterials

At present, tissue engineering has been considered as the primary therapeutic strategy for osteochondral regeneration and cartilage defect repair. However, the therapeutic effect of SF-based biomaterials on cartilage/osteochondral defects is still far from the expected effect. Consequently, it is necessary to functional modification of SF to improve the repair ability of SF-based biomaterials (**Table 1**).

#### 4.5.1. Bioactive molecules

**Figure 7:**
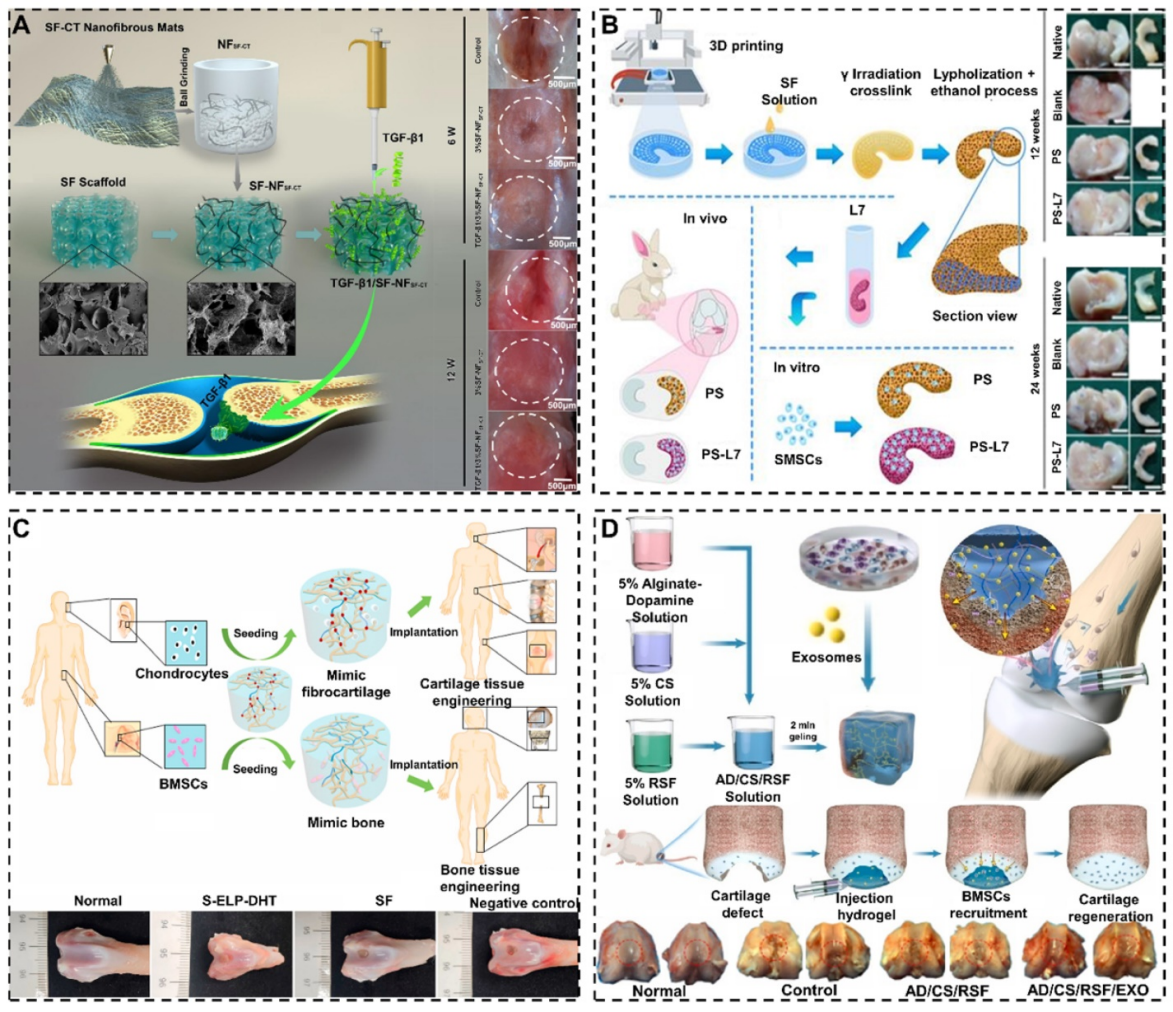
SF-based biomaterials enhance cartilage/osteochondral repair effect by functional modifications: (A) Schematic illustration of SF-NF_SF-CT_ scaffolds improve cell attachment and proliferation by hybrid treatment of SF. Adapted with permission from 177, copyright 2021, Elsevier Ltd. (B) Schematic illustration of PCL/SF scaffolds strengthen meniscus regeneration and cartilage protection by compositing with L7- peptide. Adapted with permission from 178, copyright 2020, Ivyspring International Publisher. (C) Schematic illustration of SF composite scaffolds intensifies bone repair and cartilage repair by seeding chondrocytes and BMSCs. Adapted with permission from 179, copyright 2020, Elsevier Ltd. (D) Schematic illustration of SF hydrogels enhanced cartilage repair by carrying exosomes. Adapted with permission from 180, copyright 2021, Elsevier Ltd.

It was reported that SF-based biomaterials could enhance cartilage/osteochondral repair by loading bioactive materials 181. For example, Wang et al. synthesized SF/HA scaffolds functionalized with non-immunogenic aptamer (Apt19s) 182. The Apt19s significantly improved the cell recruitment ability of SF/HA scaffolds and accelerated the repair of osteochondral. In order to promote cell adhesion, Cheng et al. hybridized SF with chitin to prepare 3D composite scaffolds (**Figure 7A**) 177. The hybrid treatment of SF formed a rough surface that well mimicked the ECM to facilitate cell attachment and proliferation. In addition, SF-based biomaterials are able to carry active molecules to treat cartilage/osteochondral defects. For example, Li et al. fabricated PCL/SF scaffolds loaded with synovium-derived mesenchymal stem cells-specific affinity peptide (L7) via 3D bioprinting technology (**Figure 7B**) 178. L7 peptide promoted the proliferation and differentiation of endogenous synovium-derived mesenchymal stem cells (SMSCs). Meanwhile, the L7 peptide stimulated the ECM production to provide a favorable microenvironment for meniscus regeneration and cartilage protection.

#### 4.5.2. Growth factors

Cartilage/osteochondral regeneration is regulated by a variety of growth factors. SF-based biomaterials loaded with growth factors can enhance the interaction with chondrocytes and osteoblasts. For example, Wu et al. fabricated biodegradable SF-gelatin porous scaffolds loading with ginsenoside Rb1 and TGF-β1 183. The results showed that the scaffolds loaded with Rb1 and TGF-β1 could create a good microenvironment for cartilage regeneration by promoting cartilage formation and inhibiting inflammation *in vivo*. Meanwhile, Chen et al. prepared SF-porous gelatin scaffolds loading with SDF-1α and TGF-β1 184. They found the scaffolds could accelerate cartilage repair by sustained release of SDF-1α and TGF-β1.

#### 4.5.3. Cells

SF-based biomaterials also could carry different cells to achieve the repair of different tissues. For example, Chen et al. designed a new type of elastin-like peptide modified SF composite scaffolds through simple and green dehydration treatment (**Figure 7C**) 179. They found that the SF composite scaffolds enhanced the adhesion, proliferation, and differentiation of BMSCs or chondrocytes *in vitro*. Additionally, the SF composite scaffolds containing BMSCs or chondrocytes could promote bone repair and cartilage repair, respectively.

#### 4.5.4. Others

In addition to bioactive molecules, growth factors and cells, loading exosomes can enhance the interaction between SF-based biomaterials and cells 185-188. For instance, Zhang et al. prepared a composite SF hydrogel loaded with BMSC derived exosomes (**Figure 7D**) 180. It was experimentally demonstrated that exosomes enhanced the recruitment of chondrocytes and accelerated cartilage repair. Furthermore, it is feasible to improve the performance of SF-based biomaterials for cartilage/osteochondral repair by loading a variety of nanoparticles (NPs) 189, 190. For instance, Li et al. prepared SF hydrogels containing BMP-2/TGF-β1@CS NPs 191. The experimental results showed hydrogels released BMP-2/TGF-β1 from CS NPs to accelerate articular cartilage repair. Meanwhile, Min et al. contrasted alginate-poloxamer/SF dual network hydrogels loading with hyaluronic acid/chitosan-poly(dioxanone) complex nanoparticles (HA/CH-PDO NPs) 192. They found that HA/CH-PDO NPs could slow-release bone morphogenic protein-7(BMP-7) to promote SMSCs differentiation into chondrocytes.

## 5. Conclusion and outlook

Cartilage and osteochondral repair have always been a clinical challenge. In recent years, tissue engineering techniques have shown certain advantages in cartilage/osteochondral repair. As a natural macromolecular material with unique mechanical properties, superior processability and excellent biocompatibility, SF is regarded as an excellent tissue engineering material. This paper reviews the research on SF-based biomaterials for cartilage/osteochondral repair. From these studies, it is concluded that SF-based biomaterials have promising applications in cartilage/osteochondral repair. For example, due to the amorphous feature of SF-based hydrogels, they could closely fit irregular cartilage/osteochondral defects 115. In addition, the controllable degradation of SF-based hydrogels enables them to adapt to different pathological conditions and provides the basis for personalized treatment. Besides, SF-based scaffolds possess more robust mechanical properties compared to other biomaterial scaffolds, which could support cell adhesion and growth better 116. In addition, the cartilage/osteochondral repair ability of SF-based biomaterials can also be improved by loading polypeptides, cells, exosomes, nanoparticles and growth factors. These functional modifications combined with bioactive substances enhance the ability of SF-based biomaterials to recruit endogenous stem cells or chondrogenesis/osteogenesis. The functional modification of SF-based biomaterials also includes the strengthening of SF itself. For example, the tyrosine-specific diazonium coupling chemistry is used to design the surface chemistry and hydrophilicity of SF for promoting the adhesion, proliferation and differentiation of BMSCs on SF-based biomaterials 193. Genetic modification of silkworms by means of gene editing can also achieve the purpose of functional modifying SF. For example, insert RGD gene sequence into mulberry silkworm SF gene to produce mulberry silkworm SF containing RGD sequence 194.

Currently, the clinical application researches (data from www.ClinicTrials.gov) demonstrate that the clinical applications of SF-based biomaterials are relatively mature in surgical sutures and wound dressings. In addition, SF-based scaffolds applied to breast reconstruction have shown remarkable improvements. It is worth noting that clinical studies on the use of SF-based scaffolds for meniscal cartilage repair have also been conducted in recent years (Table 2). Although SF-based biomaterials have promising applications, they still face several challenges in sericulture 195. On the one hand, the production standardization of cocoon (SF source) is insufficient due to the lack of international testing standards. On the other hand, the quality of silkworm cocoons is affected by several production factors, including the silkworm species, breeding method, temperature, humidity, light and hygiene conditions of the breeding environment, transportation and storage methods. Additionally, the inadequate mechanization of the sericulture industry is caused by the complex breeding conditions of silkworms and the seasonal supply of mulberry leaves. Meanwhile, the low degree of mechanization requires a lot of manual operation and leads to the high price of SF. Besides, in the aspects of SF-based biomaterials preparation, there are several factors that inhibit their clinical transformation and commercialization for cartilage/osteochondral repair. (1) The preparation process of SF-based biomaterials is tedious. The LiBr dissolution system is the most commonly used system for dissolving SF. However, the dissolution of SF by this system is time-consuming (SF is slow to dissolve and requires further dialysis, ultrasound and centrifugation after dissolution), and the obtained SF solution is insufficiently concentrated. (2) The forms and preparation methods of completed SF-based biomaterials are undiversified. (3) Most SF-based biomaterials are non-intelligent and difficult to re-intervene after implantation. (4) The entire field of cartilage/osteochondral repair is at a bottleneck stage, and it is difficult to make significant breakthroughs by only aiming to construct new SF-based biomaterials. Fortunately, some scholars have explored and discussed these limiting factors, and made certain improvements. For example, Wang et al. used CaCl_2_/formic acid solution (weight ratio of 1:20) instead of LiBr solution to dissolve SF 196. By this dissolution system, the stable and homogeneous SF solution could be obtained in 5 min. This work significantly optimized the preparation process of SF-based biomaterials. To promote the diversification of SF-based biomaterials, it is necessary to combine the advantages of various preparation methods. For instance, Chen et al. combined the advantages of electrostatic spinning, freeze-drying, and gas foaming to prepare SF-based scaffolds with excellent performance 80. Meanwhile, combining the advantages of various types of SF-based biomaterials is equally important to promote diversification. For example, Wu et al. have combined SF-based scaffolds and SF-based hydrogels to construct new SF-based composite biomaterials that integrate the advantages of scaffolds and hydrogels 144. In addition, designing SF-based biomaterials with magnetic field response is an effective way to advance the intelligence process. For instance, Wang et al. prepared a thermosensitive and pH-sensitive SF-based biomaterial that would morphological transform in alternating magnetic fields 197. Notably, Chen et al. discussed the bottleneck of bone tissue repair and the construction of bone organoids 198. Similarly, SF-based biomaterials may construct the cartilage/osteochondral-like tissues* in vitro*, which could be transplanted to achieve cartilage/osteochondral repair.

In conclusion, SF-based biomaterials have been widely studied in the field of cartilage/osteochondral repair. In the future, Researchers should develop special mechanical equipment suitable for silkworm rearing and promote mechanization to reduce the production cost of SF-based biomaterials. Meanwhile, it is also necessary to improve the preparation process of SF-based biomaterials. With the assistance of a variety of preparation methods and functional modifications, SF-based biomaterials should be more diversified and intelligent. It is important that SF-based biomaterials transform from 3D biomaterials to 4D biomaterials in combination with the fourth dimension "time". With the progress of cartilage/osteochondral repair process, 4D SF-based biomaterials can be adaptively adjusted to ensure the rapid repair of cartilage/osteochondral. We believe that SF-based biomaterials will be promising for clinical applications in cartilage/osteochondral repair.

## Figures and Tables

**Figure 3:**
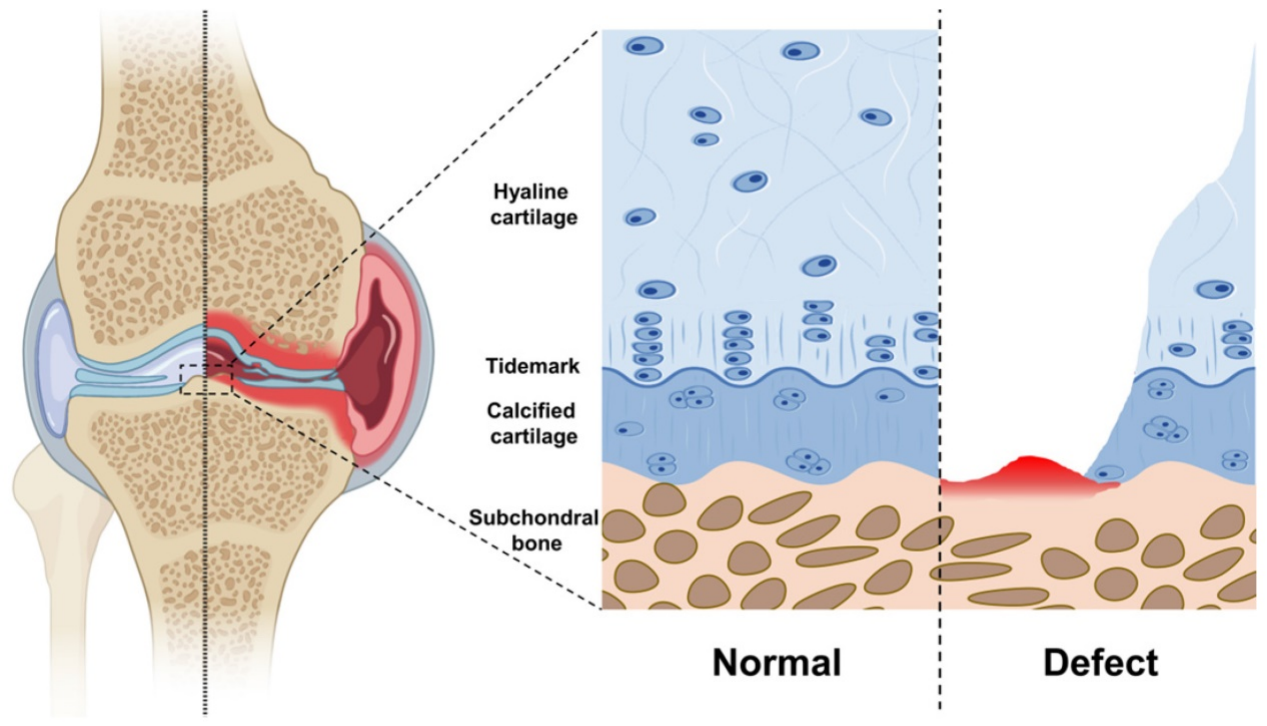
Schematic diagram of each layer of articular cartilage. Normal: From surface to interior, hyaline cartilage, calcified cartilage, and subchondral bone. The tidemark is between the hyaline cartilage and the calcified cartilage. Defect: Hyaline cartilage and calcified cartilage are worn or decomposed, subchondral bone hemorrhage.

**Table 1:** Summary of the functional modifications of SF-based biomaterials.

Types	Modifications	Functions	Reference
Bioactive molecules	E7	Promote recruit BMSCs	65, 119
	R5	Regulate osteogenic differentiation of hBMSCs	146
	L7	Promote the proliferation and differentiation of SMSCs	178
	Elastin-like peptide	Support the adhesion, proliferation and differentiation of BMSCs and/or chondrocytes	179
	Apt19s	improve the cell recruitment ability of SF/HA scaffolds; accelerate the repair of osteochondral	182
Growth factors	SDF-1	Promote the migration and differentiation of MSCs into chondrocytes	115
	SDF-1α	Enhancing cartilage forming *in vitro* and *in vivo*	184
	TGF-β1	Accelerate chondrogenesis; Stimulate cartilage and subchondral bone regeneration	90, 137, 184
	TGF-β3	Facilitate chondrocyte growth and regeneration and lateral integration with the adjoining cartilage sealed	144
	PRP	Promote the proliferation of chondrocytes	72
	PTH	Inhibit the hypertrophy of chondrocytes to maintain the phenotype of hyaline cartilage	73
	BMP-2	Stimulates the osteogenic differentiation of BMSCs	144
Cells	BMSCs	Promote bone repair	179
	Chondrocytes	Promote cartilage repair	179
	C-type natriuretic peptide gene-modified BMSCs	Accelerate the repair of articular cartilage defects	128
Others	BMP-2/ TGF-β1@CS NPs	Release BMP-2/ TGF-β1 to accelerate repair of articular cartilage	191
	HA/CH-PDO NPs	Slow-release BMP-7 to promote SMSCs differentiation into chondrocytes	192
	BMSC derived exosomes	Enhance the recruitment of chondrocytes; Accelerate cartilage repair	180

**Table 2:** Clinical application research of SF-based biomaterial.

Title	Year	Tpyes	Applications	ClinicalTrials.gov Identifier
Porous Tissue Regenerative Silk Scaffold for Human Meniscal Cartilage Repair (REKREATE)	2021	Scaffold	Meniscal cartilage repair	NCT02732873
Initial Safety Evaluation of FibroFix™ Meniscus	2017	Scaffold	Meniscal cartilage repair	NCT02205645
SeriACL™ Device Trial for Anterior Cruciate Ligament (ACL) Reconstruction	2008	Scaffold	Anterior cruciate ligament repair	NCT00490594
SERI® Surgical Scaffold Postmarket Study of Soft Tissue Support in Ventral Hernia Repair	2016	Scaffold	Soft tissue support and repair	NCT01981044
SERI® Surgical Scaffold Postmarket Study of Soft Tissue Support and Repair in Breast Reconstruction	2016	Scaffold	Soft tissue support and repair	NCT01914653
Circumferential Periareolar Mastopexy Using SERI Surgical Scaffold	2016	Scaffold	Soft tissue support and repair	NCT02293798
Clinical and Economic Outcomes With the Use of SERI® Surgical Scaffold in Direct-to-implant Breast Reconstruction	2014	Scaffold	Soft tissue support and repair	NCT02033590
Seri Surgical Scaffold Support of the Lower Pole of the Breast (SeriSupport)	2016	Scaffold	Soft tissue support and repair	NCT02016612
The SeriScaffold® Use in Reconstruction Post Market Study for Tissue Support and Repair in Breast Reconstruction Surgery in Europe	2015	Scaffold	Soft tissue support and repair	NCT01389232
SERI® Surgical Scaffold for Soft Tissue Support in Revision Augmentation Surgery	2015	Scaffold	Soft tissue support and repair	NCT02030938
Evaluation of HQ® Matrix Soft Tissue Mesh for the Treatment of Inguinal Hernia	2016	Scaffold	Treatment of inguinal hernia	NCT02487628
AUTOLOGOUS FIBRIN GLUE VERSES 4-0 SILK SUTURES IN PERIODONTAL FLAP CLOSURE	2020	Suture	Wound closure	NCT03792113
Coated VICRYL* Plus Suture Compared to Chinese Silk in Scheduled Breast Cancer Surgery	2009	Suture	Wound closure	NCT00768222
Cyanoacrylate Tissue Adhesives Versus Silk Suture at the Palatal Donor Site of Sub Epithelial Connective Tissue Graft	2021	Suture	Wound closure	NCT04780360
The Comparison of Microbial Adherence to Various Sutures in Patients Undergoing Oral Surgery	2017	Suture	Wound closure	NCT02653924
Suture Materials: an Evaluation	2016	Suture	Wound closure	NCT03410433
Clinical Evaluation of Sutures in Periodontal Surgery	2012	Suture	Wound closure	NCT02013661
Performance of Safety of SILKAM® Suture Material in Oral Surgery (SILKOS)	2022	Suture	Mucosal closure	NCT05296902
Efficacy and Safety of Silk Fibroin With Bioactive Coating Layer Dressing	2015	Wound dressing	Wound healing	NCT02091076
Evaluation of HQ® Matrix Medical Wound Dressing for Healing of Donor Site Wounds	2014	wound dressing	Wound healing	NCT01993030
Manufacturing, Characterization and Evaluation of the Effect of Silk Fibroin Membranes, Loaded or Not With Neurotensins on Open Wounds in the Palate	2022	Film	Wound healing	NCT05191082

## Abbreviations

OA: osteoarthritis
SF: silk fibroin
PLA: polylactic acid
PCL: polycaprolactone
HA: hydroxyapatite
CS: chitosan
SFG-E7: silk fibroin-gelatin-E7
BMSC: bone mesenchymal stem cell
ECM: extracellular matrix
PRP: platelet-rich plasma
PTH: parathyroid hormone
MA: methacrylic anhydride
GM: gelatin methacryloyl
3DHAS: three-dimensional/hyaluronic acid/SF scaffold
TGF-β1: transforming growth factor-β1
KGN: kartogenin
GO: graphene oxide
DCM: decellularized cartilaginous matrix
BCNR: bacterial cellulose nanoribbon
HRP-SF: horseradish peroxidase-cross-linked SF
β-TCP: β-tricalcium
BMP-2: bone morphogenetic protein-2
TGF-β3: transforming growth factor-β3
NHA: nano-hydroxyapatite
GSSR5: gradient silicified silk fibroin R5 peptide
MWNTs: multi-walled carbon nanotubes
Apt19s: non-immunogenic aptamer
NPs: nanoparticles
L7: synovium-derived mesenchymal stem cells-specific affinity peptide
HA/CH-PDO NPs: hyaluronic acid/chitosan-poly(dioxanone) complex nanoparticles
SMSCs: synovium-derived mesenchymal stem cells
LAP: laponite
BDDE: diglycidyl ether
